# Personalized Cardiovascular Regenerative Medicine: Targeting the Extreme Stages of Life

**DOI:** 10.3389/fcvm.2019.00177

**Published:** 2019-11-27

**Authors:** Paolo Madeddu, Elisa Avolio, Valeria Vincenza Alvino, Marianna Santopaolo, Gaia Spinetti

**Affiliations:** ^1^Translational Health Sciences, Bristol Heart Institute, Bristol Royal Infirmary, University of Bristol, Bristol, United Kingdom; ^2^Laboratory of Cardiovascular Research, IRCCS MultiMedica, Milan, Italy

**Keywords:** aging, regenerative, cardiovascular, stratification, personalized medicine, congenital heart disease, bone marrow, frailty

## Abstract

Cardiovascular regenerative medicine is an exciting new approach that promises to change the current care of million people world-wide. Major emphasis was given to the quality and quantities of regenerative products, but recent evidence points to the importance of a better specification of the target population that may take advantage of these advanced medical treatments. Patient stratification is an important step in drug development. Tailoring treatment to the patient's specificity allowed significant improvement in cancer therapy, but personalized regenerative medicine is still at the initial stage in the cardiovascular field. For example, new-borns with a congenital heart condition and elderly people require dedicated therapeutic approaches, which adapt to their lifetime needs. In these people, personalized treatments may overcome the benefits delivered by standard protocols. In this review, we provide insights into these extreme stages of life as potential targets for cardiovascular reconstitution.

## Introduction

Cardiovascular regenerative medicine promises to change the clinical outcome of million people. However, the translation from basic and preclinical research to the bedside has not maintained all the initial promises. Several reasons can account for these discrepancies, especially the need for further refinement of the drug or cell/gene product as well as the method and time of delivery. However, it is important to pay equal attention to the population that can take the maximum benefit from the new approach. Personalizing treatment for cardiovascular disease has had some remarkable successes in uncovering new therapeutic targets. For instance, the observation that inactivating mutations in the gene encoding the trafficking protein PCSK9 expose patients to a much lower risk for heart attacks fueled the development of antibody therapy targeting this protein. Other examples of cardiovascular drugs for which patient response is affected by the genetic makeup include warfarin and clopidogrel, used to prevent coagulation problems. A recent clinical trial with an antibody blocking the inflammatory cytokine IL-1β showed that individuals with high blood C-reactive protein (CRP) levels could take the maximum advantage for prevention of cardiovascular events (1).

Several biomarkers were proposed for improved prediction of the mode of action of stem cells in cardiac disease. These include markers of extracellular matrix remodeling, such as collagen degradation products, and inflammation, like TNF-α and CRP, which reflect continued immune dysfunction and oxidative damage in the myocardium. For instance, in the randomized TRIDENT trial (Transendocardial Stem Cell Injection Delivery Effects on Neomyogenesis), patients received transendocardial stem cell injection (TESI) of allogeneic mesenchymal stem cells (MSCs) at either a dose of 20 or 100 million cells (2). Results indicate that only those who received 100 million cells had improvements in left ventricular ejection fraction (LVEF), but both groups experienced a significant reduction in TNF-α, both in the circulation and intracellular in B-cell, indicating the immunomodulatory effects of MSCs, which may play an important role in their improving cardiac function.

The importance of pretreatment assessment of contractility markers, such as the LVEF, in influencing the outcome of cell therapy remains controversial. Two recent meta-analyses of bone marrow cell therapy trials in patients with acute myocardial infarction (MI) indicate that patients experienced similar improvement in LVEF regardless of the baseline LVEF. However, improvements in left ventricular end-systolic volume were more pronounced in patients with lower baseline LVEF. In contrast, in trials of chronic myocardial ischemia, the increase in LVEF elicited by cell therapy was significant only in the group with lower LVEF at baseline (3, 4).

Current regenerative therapies are generally delivered to middle age populations, which show heterogeneous responses to therapies. Here, we overview evidence for personalized application of cardiovascular therapeutic approaches with emphasis on the two extreme stages of life: new-born and elderly.

## Advanced Regenerative Medicine Approaches to Mend the Newborn Heart

Congenital heart disease (CHD) is characterized by an abnormality in heart structure and is the most common type of birth defect, with a reported prevalence of 9 per 1,000 births (5, 6). Despite the progress in the surgical management of patients with CHD, often solutions are temporary and only partially resolve the problem. Eventually, patients develop heart failure (HF) which contributes to high morbidity and mortality rates (7–9). In addition HF represents a major problem in the growing group of subjects that survive into adulthood, which are estimated to be 1.2 million in Europe only (10–12). HF is known to occur in ≈25% of adult CHD (ACHD) patients by the age of 30, and the incidence increases with age (13). Therefore, new therapies should be developed to integrate current approaches of corrective cardiac surgery in newborns and infants with CHD.

### Conventional Treatment of Congenital Heart Disease

The ideal cure for CHD consists of definitive surgical correction. In patients with Tetralogy of Fallot (ToF), a prototypical form of complex CHD, the aim is to relieve the obstruction to blood flow from the right ventricle (RV) to the pulmonary circulation and close the ventricular septum defect. Reconstruction of RV outflow tract (RVOT) involves resection of blocking muscle bundles and implantation of a prosthetic valve pulmonary conduit. Patients with complex CHD like TOF usually receive reconstructive surgery in infancy. Nevertheless, even full correction is not definitive. Re-interventions are necessary during a patient's life to substitute prostheses that become incompetent (14).

A spectrum of prostheses in the form of conduits, patches and valves is employed in congenital cardiac surgery, but none of them is perfect. Non-biological prostheses, like mechanical valves and Gore-Tex patches/conduits, have the advantage of high availability but do not possess growth potential (15). Moreover, mechanical valves require anticoagulation and can cause hemolysis. Biological prostheses, like autografts derived from patient's own valves/pericardium and pulmonary artery homografts from human cadavers, have excellent characteristics. However, their availability is limited. Therefore, to date, animal-derived grafts (xenografts made with bovine or swine valvular, pericardial, or intestinal material) are the most common type of biological prostheses in reconstructive cardiac surgery. However, the manufacturing process makes grafts more prone to thrombosis and degeneration (16, 17).

In recent years, the need to overcome the above-mentioned limitations paved the way to a new, exciting medical-research field, namely tissue engineering.

### New Solutions From Stem Cell-Engineering

**Figure 1:**
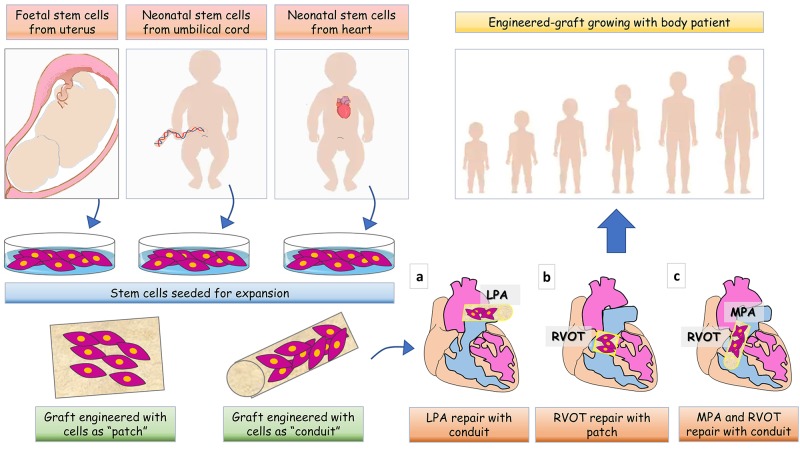
Cartoon illustrating various tissue engineering strategies for full management of patients with CHD. Based on CHD diagnosis is made pre- or post-birth, stem cells can be collected from fetal tissues, umbilical cord or leftovers from palliative cardiac surgery. After collection, cells are expanded *in vitro* and seeded in a natural scaffold to generate a shaped patch- or conduit- graft to be implanted in the heart of CHD patients. The site of implantation of the graft is showed for (a) left pulmonary artery (LPA) reconstruction, (b) right ventricle outflow tract (RVOT) reconstruction, and (c) main pulmonary artery (MPA) and RVOT reconstruction.

Landmark clinical work has demonstrated the potential of biomaterials engineered with stem cells (SCs) for definitive correction of organ defects (18, 19). The approach has been proposed to improve the durability of cardiac prostheses and thereby optimize long-term outcomes in CHD patients (20, 21). The underlying concept is that incorporation of SCs shall confer prosthetic grafts with the characteristics of a living tissue that grows in a physiologic manner in parallel with cardiac and whole body growth and withstands the impact of degeneration (Figure 1) (22, 23). Initial experimental studies focused on preventing thrombotic complications by coating prosthetic valve leaflets with autologous endothelial cells (ECs)/endothelial progenitor cells (EPCs) (24). First-in-human studies have provided initial evidence on feasibility and effective use of surface-enhanced valvular grafts (25, 26). However, to improve graft durability, additional aspects must be considered. (A) *Cell-graft interactions*. The main goals are for cells to: (i) colonize not only the surface but also the prosthesis core, (ii) survive and replicate to generate a stable resident population, (iii) dynamically synthesize ECM proteins in order to support graft stability and growth, and (iv) secrete factors favoring re-endothelialization, while preventing inflammation and calcification. These qualities are inherent to the cells, but also depend on proper interactions between the *right cell* and *right prosthesis*. Combining cells and prostheses already available in a clinical format may provide the means for swift exploitation, thus it may be advantageous to test them first. (B) *Cell potency*. Induced pluripotent SCs (iPSCs) generated by reprogramming somatic cells would be an ideal source for patient-specific therapy. However, recent reports have emphasized the pitfalls of iPSC technology, including the potential for genetic and epigenetic abnormalities, tumorigenicity, and immunogenicity (27). Hence, lineage-committed PCs remain a safer option thus far. (C) *Cell accessibility and scalability*. Thanks to advances of pre-natal cardiac imaging, it is now possible to recognize cardiac defects *in utero* and thus design tissue engineering applications for early primary correction. In this regard, fetal SCs could be obtained during an ongoing pregnancy (mid-trimester amniotic fluid or placenta specimens at the occasion of prenatal screening) or at baby delivery (placental or umbilical cord samples) (28–30).

Additionally, storage protocols of umbilical cord blood cells are well-established, thus allowing potential use for secondary correction. Recently, the Mayo Clinic announced the first trial with autologous umbilical cord blood cells to treat children with hypoplastic left heart syndrome, a defect in which the left ventricle is underdeveloped. SCs collected at birth are stored until intra-myocardial injection during secondary reconstructive surgery at 6 months of age (31).

Our group and others have reported that pericytes from human fetal hearts and aortas possess multilineage differentiation potential and vasculogenic activity *in vitro* and *in vivo* (32–34). Extending those observations, we have set up a standard operating procedure (SOP) for expansion of pericytes from remnants of neonatal surgery. We are currently proposing the novel use of autologous cardiac pericytes collected during palliative surgery for cellularization of cardiac prostheses (35).

In conclusion, in the future this novel, personalized stem cell-engineering approach promises to provide definitive solutions for the correction of CHD in the youngest patients.

## The Aging Population and the Clinical Problem of Frailty

It is worth considering that in parallel to the steps forwards in the cure of early life cardiovascular dysfunction, the same regenerative strategies that are under consideration for the middle-aged population may not be effective for the growing number of elderly people. In fact, the progresses in medicine and the social modernization/secularization, in conjunction to the decrease in birth rate concur to the aging of the world population. At present, it is estimated that 16.1% of the European population is over the age of 65 years, and this number is predicted to rise to 22% by 2031, which corresponds to approximately 137 million people (36, 37). This unprecedented demographic phenomenon is causing a great social and medical alarm, due to the expected increase of common diseases and geriatric syndromes, which often comprise more than one disorder at a time. Furthermore, many *healthy* older people become progressively unfit and incapable of handling life changes and stress, affected by the frailty syndrome. The term “geriatric frailty” was coined 30 years ago to define a clinical state in which there is an increase in an individual's vulnerability to adverse events and harm when exposed to a stressor. Primary frailty is not associated directly with a specific disease. However, in many instances, frailty is entwined with a pathological condition, like diabetes mellitus (DM) or osteoarthritis (38, 39). The Frailty Index estimates ~23% of people aged 65 or older is frail and has an increased risk of death. Furthermore, the presence of frailty increases the risk of death attributable to an associated disease. For instance, a 12-year follow-up study conducted on more than 1,200 patients showed a strong synergic effect of frailty and osteoarthritis on life expectancy (40). Frail population demands high medical and social care, absorbing a significant amount of resources from the national health systems.

### Bone Marrow Stem Cell Frailty: A Model to Reinterpret Whole Body Vulnerability

Novel experimental and clinical evidence indicates that the status of the bone marrow (BM) predicts the global outcome of vulnerable patients. The BM is the main reservoir of SCs in adulthood. We believe that its status and the function of cells released from BM into the circulation can reflect the general regenerative capacity of the human body.

Our team has demonstrated that DM, which is frequently associated with frailty (38, 39), causes a profound BM remodeling in mice and humans, with reduction of the hematopoietic tissue, microvascular rarefaction, adipose tissue accumulation, and osteoporosis. Also, we showed that hematopoietic SC (HSC) depletion was associated with increased oxidative stress, DNA damage, and activation of apoptosis (41). Furthermore, oxidative stress was responsible for an alteration of the BM vascular barrier function, contributing to stem cell mobilopathy (42). Landmark work from Fadini demonstrates that levels of circulating proangiogenic progenitor cells inversely correlate with classical cardiovascular risk factors and atherosclerotic complications in the coronary, peripheral and cerebrovascular districts. Another fundamental study in a large population with coronary artery disease showed that reduced circulating progenitor cell counts, identified primarily as CD34^pos^ cells, are associated with risk of death (43). A definitive meta-analysis of 21 studies, comprising 4,155 individuals, confirmed this association (44). Together with the notion that CD34^pos^ progenitor cells maintain cardiovascular health, these studies suggest that an impaired liberation of reparative cells from BM to the circulation contribute to promoting cardiovascular vulnerability (45). HSCs could also transfer harmful signals to the peripheral vasculature. In this way, potentially restoring responses afforded by BM-derived reparative cells could be transformed into a dangerous phenomenon. This raises the possibility of developing assays based on circulating cells for prediction of long-term cardiovascular outcomes and eventually, new therapies intercepting downstream signaling pathways. A few years ago, we started a prospective study investigating if the abundance and migratory activity of a subpopulation of circulating mononuclear cells, namely, CD45^dim^CD34^pos^CXCR4^pos^KDR^pos^ cells, predict major amputation and cardiovascular death in type 2 diabetic patients undergoing percutaneous transluminal angioplasty for critical limb ischemia. Multivariable regression model analysis at 18 months follow-up showed that *in vitro* cell migration forecasts cardiovascular mortality independently of other validated predictors, such as age, diagnosed coronary artery disease, serum CRP, and estimated glomerular filtration rate. In this model, doubling of migrated cell counts increases the cardiovascular death hazard by 100% (46). We have now confirmed the value of the predictor at 6 years follow-up and also identified a potential molecular target responsible for circulating cells to cause endothelial cell damage and death (Madeddu and Spinetti, unpublished data).

Mechanistic studies indicate that aging and DM contribute in impairing stem cell/progenitor cell mobilization *via* dysregulation of the key lifespan determinant pathway comprising the silent information regulator (SIR)T1, p66Shc, and mammalian target of rapamycin (mTOR) (47–49). These genes integrate longevity pathways and metabolic signals in a complex interplay in which lifespan appears to be strictly dependent on substrate and energy bioavailability (50). Recent data from Fadini's group indicates that cell-autonomous activation of the Oncostatin M (OSM)-p66Shc pathway leads to DM-associated myelopoiesis, whereas its transcellular hemato-stromal activation links myelopoiesis to mobilopathy. Therefore, targeting the OSM-p66Shc pathway may represent a novel strategy to disconnect mobilopathy from myelopoiesis and restore normal stem cell mobilization (51).

A large body of evidence indicates the implication of microRNAs in stem cell senescence and vulnerability. We have documented that DM remarkably alters the expression of microRNAs implicated in the control of hematopoiesis and vasculogenesis. In a cohort of subjects undergoing hip replacement for arthrosis, we showed that DM downregulates the microRNA-155 in BM HSCs, which results in induction of HSC apoptosis via induction of the target gene Forkhead Box O3a (FOXO3a) and cell cycle controllers p21 and p27kip1 (52). P21 and p27kip1 inhibit cell cycle progression by binding to, and inactivating, cyclin-dependent kinase complexes. Analysis of cell cycle by flow cytometry confirmed that CD34^pos^ cells from diabetic BM are stalled at the G1 checkpoint and undergo apoptosis with high frequency. Furthermore, we demonstrated the upregulation of several anti-angiogenic microRNAs, such as microRNA-15, 16, and 503, in circulating pro-angiogenic cells from patients with ischemic complications (52, 53).

Of note, BM HSCs accumulate mutations during aging in specific genes that lead to the generation of clonal leukocytes in the peripheral blood harboring a 2% variant allele fraction. These mutations occur in genes usually associated to acute myeloid leukemia or myelodysplastic syndrome but confer a low risk (0.5–1% per year) of developing neoplasms. This condition in the absence of morphological indication of a tumor is referred as clonal hematopoiesis of indeterminate potential (CHIP). Since CHIP carrier have an increased risk of all-cause mortality and worsen heart failure estimated to be 40%, it is clear that this risk factor will have to be taken into consideration in future patient stratification strategies in the elderly (54–56).

### Restoring BM Health in Vulnerable Individuals

Evidence from our group and others have shown that age- and DM-related BM stem cell dysfunction can be reversed by using nutritional supplements and physical exercise, both interventions exerting a salutary effect through a reduction in oxidative stress and activation of pro-survival pathways (41, 49, 57). For instance, both acute and regular exercise have been associated to increase number of circulating progenitor cells via modulation of mobilizing factors such as the duo CXCL12/CXCR4, VEGF-A, MMPs, and nitric oxide (57). Moreover, we demonstrated the feasibility of reversing the BM pathology by dietary supplementation of benfotiamine, a vitamin B1 analog and an activator of the pentose phosphate pathway, which represents a fundamental source of antioxidant equivalents and substrates for DNA synthesis and repair. These interventions are more effective if inserted in a preventive program. Therefore, recognition of early stage of frailty is of pivotal importance to avoid residual repair potential is exhausted, which may result in therapeutic failure. We are currently exploring if the assessment of BM-derived circulating proangiogenic cells could be a marker of accruing cardiovascular frailty.

Caloric restriction has been reported the most powerful intervention to retard aging and increase longevity in several species. Studies in humans have confirmed the effect of caloric restriction on the reduction of early signs of cardiovascular diseases and cognitive decline (58, 59). Indeed, caloric intake controls a broad range of functions and modifications of caloric intake elicit several systemic and cellular responses, including mitochondrial bioenergetics.

Although several signaling pathways have been associated with the effect of caloric restriction on age-associated changes and on longevity (e.g., AMPK pathway, IGF-1-like growth factor, TOR, SIRT-1, among the most known), targeting these systems did not recapitulate all the beneficial effects of diet pointing to still unknown mechanisms (60).

It will be invaluable to determine if caloric restriction exerts therapeutic benefits in frail patients.

## Future Directions

The recognition of patients' specificity for tailoring more effective regenerative treatments opens new avenues to clinical exploitation. This endeavor will be certainly helped by concomitant advances in cardiovascular imaging and mechanistic *-omics* investigation that will increase the current knowledge that we have, at least in part, described in this article. The enormous quantities of new data derived from these approaches will require increased use of computing systems. Artificial Intelligence is a technological tool capable of analyzing and inter-linking voluminous data by scanning for appropriate relationships. Companies are already training algorithms of million clinical data points from images of coronary arteries and creating intelligent platforms that can learn to detect coronary artery disease using pattern recognition. A similar approach uses coronary calcium scoring to predict a patient's risk. These platforms could be integrated with molecular data on the regenerative potential of an individual leading to the development of predictive models for personalized treatments with pharmacological rejuvenation, or exogenous application of engineered stem cells, therapeutic genes and regenerative tissue. Less invasive surgical procedures will also be key to implant advanced regenerative products improving the quality of life of the very young and elderly.

## Author Contributions

All authors listed have made a substantial, direct and intellectual contribution to the work, and approved it for publication.

### Conflict of Interest

The authors declare that the research was conducted in the absence of any commercial or financial relationships that could be construed as a potential conflict of interest.
